# Concordance of Hormone Receptor Status and BRCA1/2 Mutation Among Women With Synchronous Bilateral Breast Cancer

**DOI:** 10.3389/fonc.2020.00027

**Published:** 2020-02-11

**Authors:** Liang Huang, Qi Liu, Guan-Tian Lang, A-Yong Cao, Zhi-Ming Shao

**Affiliations:** ^1^Department of Breast Surgery, Shanghai Cancer Center/Cancer Institute, Fudan University, Shanghai, China; ^2^Department of Oncology, Shanghai Medical College, Fudan University, Shanghai, China; ^3^Department of Radiation Oncology, Shanghai Cancer Center/Cancer Institute, Fudan University, Shanghai, China

**Keywords:** BRCA1/2 mutation, hormone receptor status, synchronous bilateral breast cancer, concordance, DFS

## Abstract

**Goals:** BRCA1/2 mutations are associated with bilateral breast cancer. The extent of concordance between synchronous bilateral breast cancer (SBBC) tumors with respect to hormone receptor expression and BRCA1/2 mutations is unknown. We investigated the distribution of BRCA1/2 mutations and bilateral estrogen receptor (ER) status in SBBC.

**Methods:** A retrospective analysis was performed on 15,337 patients with primary breast cancer who underwent surgical treatment at the Fudan University Shanghai Cancer Center between 2007 and 2014. We included 163 patients with synchronous bilateral breast cancer who had germline BRCA1/2 mutations testing. BRCA1/2 pathogenic/likely pathogenic mutations and other clinicopathological characteristics were studied in further analyses.

**Results:** Patients with SBBC developed breast cancer at an older age and had a higher rate of ER positivity than patients with UBC (*p* < 0.001, separately). In contrast, 14.1% of SBBC patients had carcinomas with a lobular component in either breast based on pathological reports (*p* < 0.001). Twelve patients had BRCA1 mutations, and 14 patients had BRCA2 mutations, while no patients had mutations in both genes. The BRCA1/2 mutation rate was higher in younger patients (23.4 vs. 11.1%, *p* = 0.036). SBBC patients with a family history of breast cancer or bilateral ER-negative disease had a higher frequency of BRCA1/2 mutations than the cohort without a history of these conditions. SBBC with a bilateral ER-discordant status had a very low frequency of BRCA1/2 mutations (5.6%). Patients with an ER-positive (concordant or discordant) status had better 3-year disease-free survival than patients with a concordant ER-negative status (HR = 0.324, 95% CI: 0.126–0.837, *P* = 0.020). However, the outcomes were similar during long-term follow-up. Pathological lymph node stage was the only prognostic factor for SBBC in both univariate and multivariate Cox analyses.

**Conclusions:** Our study shows that Chinese women with SBBC have different characteristics from their UBC counterparts. SBBC patients with a younger age, family history of breast cancer, or bilateral ER-negative disease are more likely to have BRCA1/2 mutations. SBBC patients with a concordant ER-negative status had worse early outcomes. Our results suggest that there may be additional factors underlying the tumor biology and genetics of SBBC.

## Introduction

Because of the increase in breast cancer incidence rates, improvements in early diagnosis, and development of novel treatments, there is a trend of increasing occurrence for bilateral primary breast cancer. In previous reports, the incidence rate of bilateral breast cancer has ranged from 2 to 11% (1–3). Breast cancer patients are at a two to six times increased risk of developing contralateral breast tumors compared with the general population (4). Other factors for bilateral breast cancer, including family history, early age of diagnosis, lobular histology, treatment type received for the primary tumor and nulliparity, all contribute to this increased risk (1, 5–7). Most metachronous tumors are diagnosed during long-term follow-up. Synchronous tumors are less frequent, although their incidence may be increasing with modern imaging techniques. Synchronous and metachronous bilateral invasive breast cancers have different characteristics and outcomes (8).

BRCA1 and BRCA2 are located on 17q21 and 13q12, respectively. A large number of previous studies have reported BRCA1/2 as the most important tumor suppressor gene associated with breast cancer and ovarian cancer. Women with breast cancer and a BRCA mutation have a high risk of developing contralateral breast cancer. The histopathologic characteristics and the biologic behavior of SBBCs are still unclear. The relationship between hormone receptor status and BRCA1/2 mutation is also unclear. In addition, whether hormone receptor status and BRCA1/2 mutation predict worse clinical outcomes among SBBC patients remains to be determined. The aims of this study were to determine the concordance rate between hormone receptor status and BRCA1/2 mutations in women with SBBC and to identify clinical, pathologic features, and genetic predictive factors in this population.

## Methods

### Study Population

The study cohort in the present study was selected consecutively from patients who were diagnosed and treated at the Fudan University Cancer Center from 2007 to 2014. Data were gathered from patient medical records and included information from clinical, imaging, and pathology studies. Those who had bilateral breast surgery were identified as *in situ* or invasive carcinoma in bilateral breasts diagnosed within 12 months. Patient demographics and tumor variables were obtained from medical records and the hospital's electronic database. The variables analyzed included age, year of diagnosis, age at menarche, family history, BMI, number of pregnancies, and pathological features. Patients who were diagnosed with female unilateral breast cancer (UBC) during the same time period served as the control group.

### BRCA1/2 Testing

One hundred and sisty-three SBBC patients were tested for BRCA1/2 germline mutations. All coding regions and exon-intron boundaries of the BRCA1/2 genes were screened. The intronic sequence length was 70 bp on average (ranging from 5 to 204 bp). The detailed procedures of NGS and interpretation of the mutations are described in a previous article published by our institution (9). Those genetic variants that were defined as pathogenic/likely pathogenic were selected for further analysis. However, the present study did not include large genomic rearrangements (LGRs) in BRCA1/2. Sanger sequencing was used for all mutations considered to be disease associated. The sequencing results were compared with the BRCA1 (NM_007294.3) and BRCA2 (NM_000059.3) reference sequences for variant detection. The study was conducted according to the principles expressed in the Declaration of Helsinki and was approved by the institutional review board of Fudan University Shanghai Cancer Center. All patients enrolled in this study voluntarily provided written informed consent.

### Statistics

Fisher's exact test and chi-square tests were used to evaluate differences in the clinicopathological characteristics, such as BRCA1/2 mutation frequency across groups and ER status. Univariate and multivariate survival analysis was performed using the Cox proportional hazards models Disease-free survival (DFS) was calculated from the date of first diagnosis to the date of disease relapse or metastasis. Overall survival (OS) was calculated from the date of first diagnosis to the date of death or last follow-up. All analyses were performed using SPSS software version 19.0 (IBM Institute, Chicago, IL, USA). All *P*-values were two-sided, and *P* < 0.05 was considered statistically significant.

## Results

### Differences in the Clinicopathological Characteristics of Patients With SBBC and UBC

A total of 15,337 patients with primary breast cancer underwent surgery at the Fudan University Shanghai Cancer Center between 2007 and 2014. One hundred and sixty-three SBBC patients with germline BRCA1/2 testing and integral clinicalpathological information in the same period was included. Patients treated for metachronous bilateral breast cancer (MBBC), male patients and stage IV patients who underwent palliative operations during the same time period were excluded from the study. Locally advanced patients, such as those with stage IIIb or IIIc disease, were excluded from the present study to avoid the risk of misclassifying metastatic bilateral breast cancer.

As shown in Table 1, patients with SBBC developed malignant carcinoma at an older age than patients with UBC (median age: 54 vs. 51 years, *P* < 0.001). Compared with the 9.6% of UBC patients who had a family history of breast cancer, 11.7% of patients with SBBC had a similar family history within first-degree relatives. In contrast, 14.1% of SBBC patients had carcinomas with a lobular component in either breast based on pathological reports (*P* < 0.001). There was no significant difference in TNM stage distribution. However, SBBC patients tended to have received bilateral mastectomy, and only 8.0% of SBBC patients chose breast-conserving surgery or breast reconstruction compared with 24% of UBC patients (*P* < 0.001). ER-positive carcinoma was more often found in SBBC patients than in UBC patients (87.1 vs. 74.0%, *P* < 0.001), and HER2 status was similar in both groups.

**Table 1:** Demographics and clinicopathological characteristics of SBBC and UBC patients.

**Variables**	**SBBC (*n* = 163)**	**UBC (*n* = 13,832)**	
Age (median, range)	54 (26–84)	51 (18–98)	*P* < 0.001
Family history	19 (11.7%)	1,333 (9.6%)	*P* = 0.386
**Histopathology**			
No lobular component	140 (85.9%)	13,615 (98.4%)	*P* < 0.001
Lobular component	23 (14.1%)	217 (1.6%)	
**Stage**			
0/I	66 (40.5%)	5,451 (39.4%)	*P* = 0.779
II/III	97 (59.5%)	8,381 (60.6%)	
Type of surgery			*P* < 0.001
Mastectomy	150 (92.0%)	12,018 (78.4%)	
Breast-conserving surgery or breast reconstruction	13 (8.0%)	3,319 (21.6%)	
ER positivity	142 (87.1%)	6,384/8,629 (74.0%)	*P* < 0.001
HER-2-positive invasive carcinoma	31 (19.0%)	1,960/9,410 (20.8%)	*P* = 0.599

*SBBC, synchronous bilateral breast cancer; UBC, unilateral breast cancer; ER, estrogen receptor*.

### BRCA1/2 Mutation Status in Patients With Synchronous Bilateral Breast Cancer

A total of 163 SBBC patients underwent germline testing for BRCA1/2 mutation after surgery. Twenty-six patients had pathogenic/likely pathogenic mutations in either BRCA1 or BRCA2. Out of 64 early-onset (<50 years) SBBC patients, 15 BRCA1/2 mutation carriers were identified. The BRCA1/2 mutation rate was higher in younger patients (23.4 vs. 11.1%, *P* = 0.036). Seven carriers were found among 18 patients with a family history of breast cancer, which was significantly more frequent than the incidence in the cohort without a family history of breast cancer (38.9 vs. 11.3%, *P* = 0.005). SBBC patients had a higher incidence of lobular carcinoma than UBC patients, while there was no relationship between lobular carcinoma and BRCA1/2 mutation, as shown in Table 2. No significant relationship was observed between pathological T/N stage and BRCA1/2 mutation.

**Table 2:** Comparison of clinicopathological characteristics between BRCA1/2 status and ER IHC status.

**Category**		**BRCA1/2 status**			**ER IHC status**		
		**Wild type (*n* = 137)**	**Mutation status (*n* = 26)**	***P*-value**	**Bilateral concordant (*n* = 127)**	**Bilateral discordant (*n* = 36)**	***P*-value**
Age at menarche	≤ 14 years	60	13	0.56	57	16	0.963
	>14 years	77	13		70	20	
Menopause status	Premenopausal	50	12	0.352	52	10	0.151
	Menopausal	87	14		75	26	
Age	≤ 50 years	49	15	0.036	56	8	0.018
	>50 years	88	11		71	28	
Family history	Breast cancer	11	7	0.005	14	4	0.988
	No history	126	16		113	32	
pT	pTis/pT1	54	12	0.521	52	14	0.824
	pT2/pT3	83	14		75	22	
BMI	<24	91	18	0.78	92	17	0.005
	≥24	46	8		35	19	
Number of pregnancies	≤ 2 times	77	11	0.192	65	23	0.177
	>2 times	60	15		62	13	
pN	LN-negative	98	14	0.075	89	23	0.48
	LN-positive	39	12		38	13	
Histopathology	Lobular component	21	2	0.537	19	4	0.558
	No lobular component	116	24		108	32	
Invasive component	Bilateral invasive carcinoma	78	17	0.652	73	22	0.909
	Unilateral invasive carcinoma	44	6		40	10	
	Bilateral carcinoma *in situ*	15	3		14	4	
ER status	Bilateral positive	89	17	0.022	–	–	
	Bilateral discordant	34	2		–	–	
	Bilateral negative	14	7		–	–	
BRCA1/2 status	Wide type	–	–		103	34	0.07
	Mutation type	–	–		24	2	

*pT, pathological tumor stage; pN, pathological lymphnode stage; BMI, Body Mass Index*.

Twelve patients had BRCA1 mutations, and 14 patients had BRCA2 mutations, while no patients had mutations in both genes. In bilateral ER-negative patients with a family history of breast cancer, the BRCA1 mutation rate was nearly 60%. All patients with BRCA2 mutations had ER positivity on one or both sides, while 41.7% of BRCA1 mutation carriers had ER-positive disease. BRCA1 mutation was closely associated with the occurrence of ER-negative breast cancer (*P* = 0.001). DCIS with/without invasive cancer had no relationship with BRCA1/2 mutation or concordance ER status.

### Evaluation of Possible Clinical and Pathological Risk Factors for SBBC

When patients were divided into bilateral ER-positive, bilateral ER-negative and bilateral ER- discordant disease subgroups, the BRCA1/2 mutation was highest in the bilateral ER-negative subgroup (33%, *P* = 0.022). Synchronous bilateral breast cancer patients with bilateral ER inconsistency had a very low frequency of BRCA1/2 mutations (5.6%). Patients with a high BMI (BMI ≥ 24) were more likely to have the luminal type of bilateral breast cancer (*P*= 0.005). Follow-up data were available for all 163 invasive breast cancer patients in our cohort. The median follow-up was 56.1 months (range: 21.0–139.8 months). No significant differences were observed in disease-free survival (DFS) between the BRCA1/2 carriers and non-carriers [hazard ratio (HR) = 1.068, 95% confidence interval (CI): 0.409–2.790, *P*= 0.894], BRCA1 carriers and non-carriers (HR = 1.506, 95% CI: 0.457–4.967, *P* = 0.501), or BRCA2 carriers and non-carriers (HR = 0.730, 95% CI: 0.174–3.067, *P* = 0.668). There was also no significant difference in overall survival (OS) between BRCA1/2 carriers and non-carriers [hazard ratio (HR) = 1.229, 95% confidence interval (CI): 0.264–5.727, *P* = 0.793].

**Figure 1:**
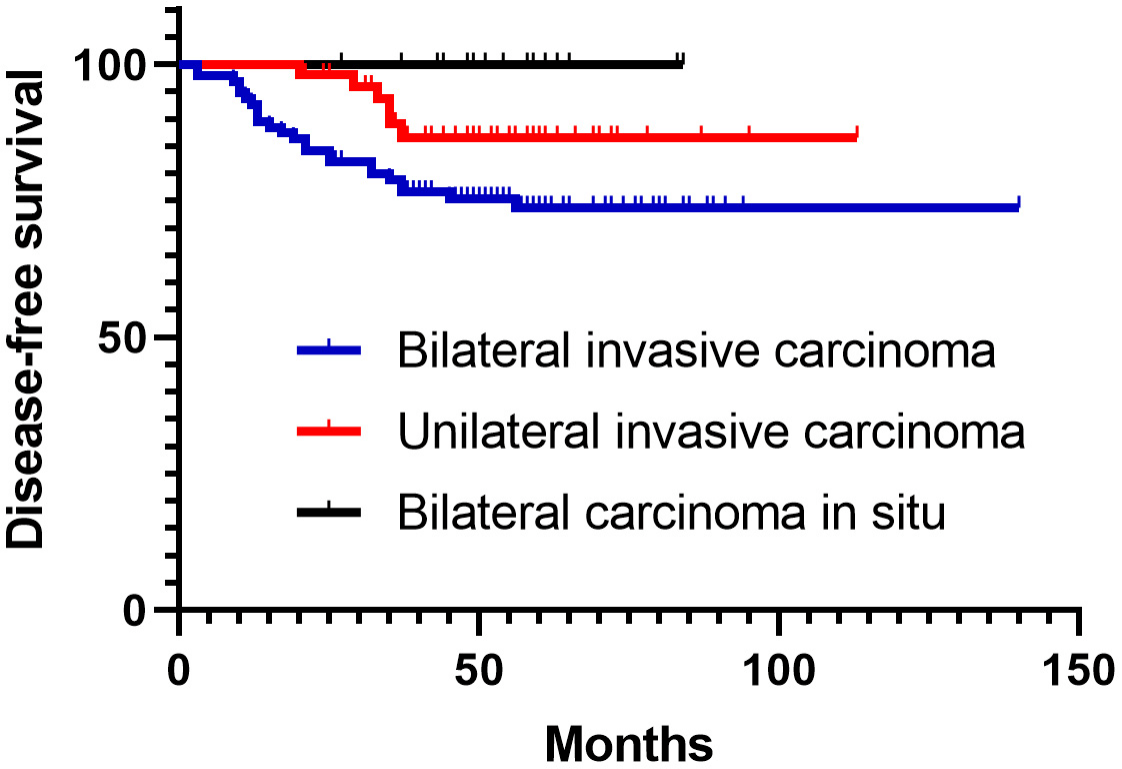
Log-rank curve for DFS among bilateral carcinoma *in situ*, unilateral invasive carcinoma and bilateral invasive carcinoma groups.

No significant difference was observed in relation to other clinical and pathological factors (age at menarche, menopause status, age, family history, pathological tumor stage, BMI, number of pregnancies, lobular carcinoma, bilateral ER consistency, ER status, and LVI). Patients with an ER-positive (concordant or discordant) status had better 3-year disease-free survival than patients with a concordant ER-negative status (HR = 0.324, 95% CI: 0.126–0.837, *P* = 0.020). However, this difference disappeared during long-term follow-up. Concordance and discordance of HER2 status was no relationship with BRCA1/2 mutations and survival. Pathological lymph node stage was the only prognostic factor for synchronous bilateral breast cancer in both univariate and multivariate Cox analyses (*P* = 0.001 and *P* = 0.01), as shown in Table 3. SBBC patients were divided into pure bilateral carcinoma *in situ*, unilateral invasive carcinoma and bilateral invasive carcinoma groups. The bilateral invasive carcinoma patients had the worst DFS, which was showed in Figure 1 (*P* = 0.026). There was no difference in OS analysis.

**Table 3:** Univariate and multivariate survival analyses of SBBC patients.

**Category**		**Univariate Cox analysis (DFS)**		**Multivariate Cox analysis (DFS)**	
		**Hazard ratio (95% CI)**	***P*-value**	**Hazard ratio (95% CI)**	***P*-value**
Age at menarche	≤ 14 years vs. >14 years	1.696 (0.823–3.491)	0.152	–	–
Menopause status	Premenopausal vs. menopausal	1.282 (0.623–2.641)	0.5	–	–
Age	≤ 50 years vs. >50 years	0.832 (0.404–1.715)	0.619	0.887 (0.391–2.010)	0.774
Family history	Breast cancer vs. no history	0.556 (0.212–1.454)	0.231	–	–
pT	pTis/pT1 vs. pT2/3	1.966 (0.875–4.416)	0.102	1.027 (0.431–2.448)	0.952
BMI	<24 vs. ≥24	1.236 (0.588–2.600)	0.576	–	–
Number of pregnancies	≤ 2 times vs. >2 times	1.758 (0.823–3.757)	0.145	–	–
pN	LN-negative vs. LN-positive	0.291 (0.141–0.599)	0.001	0.361 (0.166–0.787)	0.01
Histopathology	Lobular component vs. no lobular component	0.730 (0.279–1.907)	0.521	0.746 (0.252–2.203)	0.595
Bilateral ER consistency	Bilateral concordant vs. bilateral discordant	0.951 (0.409–2.217)	0.907	–	–
ER status	Positive vs. negative	0.506 (0.207–1.240)	0.136	0.473 (0.167–1.338)	0.158
HER2 status	Positive vs. negative	0.614 (0.263–1.430)	0.258	0.531 (0.208–1.357)	0.186
BRCA1/2 status	Wild type vs. mutated	1.068 (0.409–2.790)	0.894	0.585 (0.431–2.448)	0.355
LVI	Positive vs. negative	0.664 (0.304–1.450)	0.304	–	–
ER status for 3y DFS	Positive vs. negative	0.324 (0.126–0.837)	0.020	0.179 (0.050–0.644)	0.008

## Discussion

The incidence of synchronous bilateral invasive breast cancer ranges from 0.3 to 3.0%. Differing definitions have been used for a diagnosis of synchronous disease, with cut-offs ranging from within 3 to 12 months of initial diagnosis (10, 11). Some previous studies have determined that the age at diagnosis of the initial tumor is an important predictor for contralateral breast cancer. In our study, the average age at diagnosis of SBBC was 54 years old, which was significantly older than the age (51 years) observed for UBC. However, Chinese SBBC patients were still younger than Caucasian patients (12). A previous study also showed that the survival rate in patients with bilateral breast cancer is significantly lower than the survival rate in patients with UBC (13, 14). However, a small study also reported that there were no significant differences in disease-specific survival between SBBC patients and UBC controls (12). Due to the lower incidence and variable definitions of SBBC, the survival rates differ. In previous research, incidence and outcome were markedly different between MBBC and SBBC. SBBC, compared to MBBC and UBC, had a significantly higher distant metastasis rate. SBBC also had worse disease-specific survival and OS than MBBC (8). Lobular histology, family history and BRCA mutations have been associated with an increased incidence of bilateral presentation (15). Our study described the clinicopathologic features of SBBC in a large cohort of patients consecutively treated in a single institution and compared these characteristics with those of a large cohort of patients with unilateral invasive breast carcinoma treated during the same time period. As we confirmed in our study, SBBC patients were older than UBC patients and showed a higher incidence of lobular carcinoma, a higher rate of ER-positive status and a higher rate of mastectomy. Bilateral tumors may have different pathological features. The concordance between MBBC for ER status was much greater than expected for BRCA1/2 mutation (16). Several studies in predominantly symptomatic patients found that BBCs show more favorable tumor characteristics than unilateral cancers, with a higher proportion of invasive lobular cancer, a lower T stage and a higher proportion of hormone receptor positivity (12, 17).

BRCA1/2 mutations are associated with a high risk of developing contralateral breast cancer by the age of 70 years (18–21). One study found that the incidence of contralateral breast cancer was four to five times higher than that of sporadic breast cancer in BRCA gene mutation carriers, while another study found that the frequencies of BRCA mutations between bilateral and unilateral breast cancer were not different (22, 23). The prevalence of BRCA mutations in Asian bilateral breast cancer ranges from 6 to 16% (23, 24). Some studies have reported a significant correlation between family history and SBBC; however, this has not been confirmed by others (25). Interactions between genetic and hormonal environmental factors play a crucial role in the development of both unilateral and bilateral breast cancer. The most important genetic factors are BRCA1 and BRCA2. In our SBBC patients, bilateral ER-negative status, young age and a family history of breast cancer were associated with a high risk of BRCA1/2 mutation.

Tumor heterogeneity is becoming important in the management of breast cancer. In the neoadjuvant setting, a positive-to-negative change in hormone receptor status was an independent survival predictor (26). A worse prognosis was also found to be associated with a discordant receptor status between the metastatic site and the primary breast cancer (27). SBBC has been shown to have a high rate (77–88%) of concordant ER positivity (12, 28, 29). Phenotypically synchronous tumors have been shown to have a greater degree of concordance than phenotypically metachronous tumors (30). The rate of discordant ER expression between primary and secondary cancer has been shown to be as high as 29–37% in MBBC (29, 31). Some researchers have suggested that MBBC may have different genetic signatures and origins. Some oncologists retrospectively analyzed the heterogeneity of molecular markers in multifocal/multicentric breast cancer, leading to changes in adjuvant treatment in 12% of cases (32). Even in estrogen receptor-positive, HER2-negative, node-negative, synchronous bilateral invasive breast cancer, Oncotype DX risk group was discordant in 33% of women, which led to a change in treatment in 57% of these patients (33).

A meta-analysis including 8,050 patients with SBBC, defined as a contralateral diagnosis within 6 months, reported a worse prognosis for women with SBBC than that for patients with unilateral disease (34). The idea that patients with SBBC have a worse prognosis was widely adopted according to the concept that patients with tumors with worse prognostic factors need more extensive surgical treatment (35, 36). Based on SEER data, oncologists found that heterogeneity in hormone receptor status could be used to predict the overall survival and breast cancer-specific survival in the first 5 years. The ER status had a better prognostic value than the PR status (31). The hormone receptor statuses of both tumors were recorded in this study to explore the relationship between outcome and receptor variation. Our findings support the need to evaluate bilateral hormone receptor expression. In our research, we found that bilateral ER-negative patients have higher instances of short-term recurrence and metastasis, especially in the first 3 years. The bilateral ER-positive or ER-discordant patients in our cohort also had similar outcomes. In a previous report featuring Caucasian patients, there was no statistically significant difference between the different ER expression groups in either OS or BCSS after 5 years of follow-up (32).

A potential limitation of our study might be the small number of women with SBBC included. Our study also has limitations because of its retrospective character. Furthermore, patients received different chemotherapy regimens. The survival difference between ER bilateral negative and ER-positive patients in short follow-up may be statistical significance is due to chance. The strengths of our study include (1) the large population-based cohort, (2) the thoroughness, long follow-up and BRCA1/2 mutation testing, (3) uniformity in treatment location, and (4) uniformity in the pathology laboratory used. Future randomized studies with larger prospective cohorts and longer-term follow-up are needed to validate these findings.

## Data Availability Statement

The raw data supporting the conclusions of this article will be made available by the authors, without undue reservation, to any qualified researcher.

## Ethics Statement

The studies involving human participants were reviewed and approved by the study was conducted according to the principles expressed in the Declaration of Helsinki and was approved by the institutional review board of Fudan University Shanghai Cancer Center. The patients/participants provided their written informed consent to participate in this study.

## Author Contributions

LH and A-YC conceived and designed the study. Z-MS provided these clinical cases. LH and QL sorted and analyzed the clinicopathological data. LH and G-TL prepared the tables. A-YC wrote the main manuscript.

### Conflict of Interest

The authors declare that the research was conducted in the absence of any commercial or financial relationships that could be construed as a potential conflict of interest.
